# Using affected embryos to establish linkage phase in preimplantation genetic testing for thalassemia

**DOI:** 10.1186/s12958-022-00948-9

**Published:** 2022-04-30

**Authors:** Zhanhui Ou, Yu Deng, Yunhao Liang, Zhiheng Chen, Ling Sun

**Affiliations:** Center of Reproductive Medicine, Guangzhou Women and Children’s Medical Center, Guangzhou Medical University, Guangzhou, 510623 Guangdong China

**Keywords:** Preimplantation genetic testing, α-Thalassemia, β-Thalassemia, SNP linkage, Next-generation sequencing, Affected embryo, Monogenic diseases

## Abstract

**Background:**

This study aimed to evaluate the ability of next-generation sequencing (NGS) to conduct preimplantation genetic testing (PGT) for thalassemia using affected embryos.

**Methods:**

This study included data from 36 couples who underwent PGT for thalassemia without probands and relative pedigrees. NGS results were compared with prenatal diagnosis results.

**Results:**

Thirty-six couples (29 α-thalassemia and 7 β-thalassemia) underwent 41 PGT cycles (31 α-thalassemia and 10 β-thalassemia). Analysis using NGS produced conclusive results for all biopsied blastocysts (100%, 217/217). One hundred and sixty (73.7%, 160/217) were unaffected by thalassemia. Preimplantation genetic testing for aneuploidy revealed that 112 (70.0%, 112/160) were euploid. Single blastocysts were transferred into the uteri of 34 women (53 frozen embryo transfer [FET] cycles). Thirty-two cycles resulted in clinical pregnancies, with a clinical pregnancy rate of 60.1% (32/53) per FET cycle. Twenty-two cycles (22 couples) resulted in 23 live births, with a live birth rate of 43.4% (23/53; 3 cycles were ongoing pregnancies). All 25 embryos’ prenatal diagnosis results and/or thalassemia gene analyses after delivery were concordant with the NGS-PGT results. Seven embryos (21.9%, 7/32) were miscarried before 12 weeks’ gestation, and the abortion villus in four showed a normal karyotype and thalassemia results consistent with the NGS-PGT results. Aborted fetus samples from 3 cycles were not available because the pregnancy lasted less than 5 weeks.

**Conclusion:**

NGS can be used to conduct PGT for thalassemia using affected embryos as a reference.

**Trial registration:**

Retrospectively registered.

**Supplementary Information:**

The online version contains supplementary material available at 10.1186/s12958-022-00948-9.

## Capsule

Thirty-six couples successfully underwent PGT for thalassemia through NGS using affected embryos as a reference.

## Introduction

Thalassemia, one of the most common monogenic diseases, is an inherited blood disorder characterized by a reduction in the synthesis of hemoglobin (HB) subunit α or β (HB α or β chain). In southern China, the prevalence of α-thalassemia and β-thalassemia carriers is 8.53 and 2.54%, respectively [1]. This genetically inherited disease has threatened the lives of millions of people for decades, and no effective treatments are available. Homozygotes with the Southeast Asian (SEA) deletion develop Hb Bart’s hydrops fetalis syndrome resulting in mortality either late in gestation or soon after birth [2]. Infants with severe β-thalassemia can now survive but require extensive medical care, resulting in a rising global economic and healthcare burden [3].

Prenatal diagnosis is advocated in China to prevent the birth of babies with severe thalassemia. However, it is an invasive procedure that may induce miscarriage and may burden parents with the termination of an affected pregnancy. At present, preimplantation genetic testing for monogenic disease (PGT-M) can effectively prevent thalassemia in the children of couples who are at risk of transmitting this genetic condition to their offspring [4, 5].

Since the early 1990s, PGT-M has been used for X-linked genetic diseases [6], with PCR-based methods being used in past decades. However, allele dropout (ADO) is a main cause of misdiagnosis in PGT-M, so a direct PCR approach cannot be used as the sole method for diagnosis and detection of the target pathogenic mutation sites [7]. In recent years, linkage analysis has been widely used to increase PGT-M accuracy [8]. This method relies on short tandem repeat (STR), or single nucleotide polymorphism (SNP) markers linked to the mutations, but in some cases with de novo mutations or a lack of a proband, haplotypes cannot be constructed.

Consequently, some inherited genetic diseases, like α-thalassemia, are difficult to detect with PGT. Detection of α-thalassemia is particularly important as babies with this condition usually decease immediately after birth. Sometimes couples discover their status as carriers in the preoperative examination before in vitro fertilization (IVF) treatment. Thus, it is particularly important to construct haplotypes for thalassemia. In recent years, constructing the haplotypes through both parents has been a common approach, but this method is tedious and expensive. Another method is through single sperm and polar body diagnosis using NGS when lacking relatives or a proband, which is also used in clinics [9, 10]. However, this method requires an extra biopsy to collect polar bodies, and often needs multiple single sperm cells, which is tedious and expensive. Recently, some studies have explored constructing the haplotypes using affected embryos with NGS [4, 11, 12]. However, since these studies included only one or two cases, this method requires further validation.

In this study, we conducted PGT for thalassemia using SNP haplotyping with affected embryos as a reference. To the best of our knowledge, this is the largest study to use PGT-M for thalassemia without the relatives and probands in a clinic setting.

## Materials and methods

### Patients

This study was approved by the Reproductive Medical Ethics Committee of Guangzhou Women and Children’s Medical Center. Thirty-six couples where both parents carried genes for either α-thalassemia (29 couples) or β-thalassemia (7 couples) were selected at the Guangzhou Women and Children’s Hospital between June 2017 and June 2021. Written informed consent was obtained from each couple.

### Blastocyst biopsy and vitrification

We performed standard ovarian stimulation, intracytoplasmic sperm injection (ICSI), embryo culture, and blastocyst vitrification for each of the 36 couples as previously reported [12, 13]. Biopsy was performed on day five or six, depending on the blastocyst grade on the day of biopsy [7].

### Whole-genome amplification (WGA)

Multiple displacement amplification (MDA) using a REPLI-g Single Cell Kit (Qiagen, Germany) was performed according to the manufacturer’s protocol. The WGA products were purified and then sequenced. All PCR amplifications were performed on a 96 Well Thermal Cycler Veriti DX (Life Technologies). All procedures were carried out in accordance with the manufacturer’s protocol, as previously reported [12].

### PGT-M validation

The β-thalassemia mutation site of the WGA products was detected using 2× GoldStar Best MasterMix (Dye) (CoWin Biosciences). Primers were designed using Primer 5.0 software. Sanger sequencing was performed on an Applied Biosystems 3500 platform (Life Technologies) after PCR amplification was conducted. All of the procedures were carried out in accordance with the manufacturer’s protocols.

The α-thalassemia mutation site was detected using conventional PCR. To identify normal a-globin and SEA-type deletion alleles, WGA products from all biopsy samples were amplified with a nested PCR protocol and second PCR reaction. The PCR protocol and primers were carried out in accordance with a previous study [14].

### NGS sequencing and haplotype construction

Next-generation sequencing was performed for both SNP haplotyping and mutation locus analysis, employing hundreds of primer pairs. Mutation and SNP sites were submitted to Ion Ampliseq Designer (https://www.ampliseq.com/) for primer design. Overall, 138 SNPs within 1 Mb upstream and 132 SNPs within 2 Mb downstream of the mutation gene (chr16:215400–234,700 NM_000517.4 (HBA2) and NM_000558.4 (HBA1)) were selected for NGS-based α-thalassemia SNP haplotyping. Eighty-five SNP markers located either 1 Mb upstream or downstream of the mutation gene (chr11:5246696–5,248,301 NM_000518.4 (HBB)) were selected for NGS-based β-thalassemia SNP haplotyping. Only the SNPs that were heterozygous in one parent and homozygous in their parent were considered as informative SNPs. The genomic DNA of the couple and the WGA products were amplified with these primers for haplotype construction. Sequencing libraries were prepared using the sequencing library kit (NEXTflex Rapid DNA-seq Kit 96rxns, BIOO), and the libraries were sequenced on an Illumina MiseqDX platform (Illumina) using a MiSeq Dx Reagent Kit V3 (Illumina). All procedures were carried out in accordance with the manufacturer’s protocol. The sequencing data were analyzed by Peking Jabrehoo Med Tech., Ltd.

### Copy Number Variations (CNV) analysis

Copy number variations analysis for aneuploidy testing was performed as previously described [13]. In brief, the Illumina MiSeq platform was used for NGS, and approximately 1.5 million fragments of amplified DNA from each TE biopsy were sequenced. After the low-quality bases and adaptors were removed, clean and high-quality reads were compared with the hg19 reference genome. Unique mapped reads were calculated, and a reference dataset was obtained to represent the relative copy number. The PGXcloud cloud server (available at http://www.pgxcloud.com/) was used to analyze the chromosomal CNVs (Jabrehoo, China).

### Frozen embryo transfer and follow up

Hormone replacement therapy was used to prepare the uterine endometrium. A frozen non-pathogenic blastocyst with euploid karyotype was thawed and cultured for 2 hours before being transferred into the uterus as previously described [15]. Clinical pregnancies (CP) were defined after observation of a gestational sac with or without a fetal heartbeat on ultrasound evaluation 4 weeks after frozen embryo transfer (FET). Clinical miscarriage was determined to occur when a pregnancy failed to progress after an intrauterine gestational sac had been detected with pelvic ultrasonography. Amniocentesis was performed at approximately 17 weeks of gestation and/or gene detection of the blood was performed after birth to verify consistency with the PGT results.

### Statistical analysis

Statistical analysis was performed using SPSS software v. 19 for Windows (SPSS Inc., Chicago, USA), applying parametric and nonparametric tests where appropriate. Continuous variables were expressed as means ± standard deviations (SD) and analyzed using the Student’s t-test. Categorical variables were expressed as percentages and analyzed using χ^2^ or Fisher’s exact test depending on the sample size. Statistical significance was defined as *p*-values less than 0.05.

## Results

### Trophectoderm biopsy and WGA

**Fig. 1:**
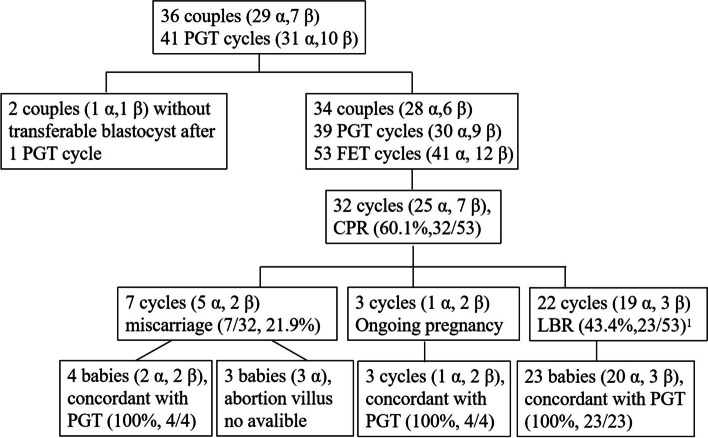
Flow chart and pregnancy outcomes of the study. ^1^Family 15 developed a monochorionic diamniotic twin pregnancy after a single blastocyst transfer, resulting in the birth of two healthy babies. Α: α-thalassemia, β: β-thalassemia

The 36 couples (29 α-thalassemia and 7 β-thalassemia) underwent a total of 41 PGT cycles (31 α-thalassemia and 10 β-thalassemia) (Table 1 and Supplementary Table 1) (Fig. 1). Seven hundred and seventy-seven oocytes were retrieved. Of these, 631 were fertilized with intracytoplasmic sperm injection (ICSI), and 217 were cultured to blastocysts of good enough quality to perform trophectoderm (TE) biopsy (better than IIICC). The average number of blastocysts for each couple was 6.0 (217/36). Whole-genome amplification was successfully performed on all TE cells. The allele amplification rate was 94.71%, and the ADO rate was 4.26%.

**Table 1:** The preimplantation genetic testing outcomes of the 36 families

Family	Cycles		Embryo state						PGT results		Pregnancy results		Genetic testing		
	PGT	FET	Oocyte	MII	2 PM	Cleavage	Blastocyst	Biopsy blastocyst	Unaffected^a^	Transferrable^b^	CP	LB	PGT-M	Amniocentesis	After born
1	1	1	26	25	18	17	12	9	6	3	1	1	Wild type	Wild type	Wild type
2	1	1	22	17	15	15	8	8	6	4	1	1	Heterozygote	Heterozygote	Heterozygote
3	1	1	13	12	10	10	6	3	2	1	1	1	Wild type	NA	Wild type
4	1	1	11	10	9	9	7	5	2	2	1	1	Wild type	NA	Wild type
5	1	1	12	12	11	11	11	9	6	3	1	1	Heterozygote	Heterozygote	Heterozygote
6	1	2	15	14	12	11	10	7	6	2	1	1	Heterozygote	Heterozygote	Heterozygote
7	1	1	24	20	15	14	8	4	4	1	1	1	Heterozygote	Heterozygote	Heterozygote
8^c^	2	2	44	31	21	21	15	8	6	4	1	1	Heterozygote	NA	Heterozygote
9	1	2	35	27	21	21	16	6	6	6	1	0	Wild type	Miscarriage	NA
10	1	1	28	16	11	11	7	4	1	1	1	0	Wild type	Miscarriage	NA
11	1	1	15	11	10	10	6	5	3	2	1	1	Wild type	Wild type	NA
12	1	3	21	16	13	13	7	4	4	3	1	1	Heterozygote	NA	Heterozygote
13	1	2	26	20	16	16	13	9	7	5	1	1	Wild type	Wild type	NA
14	1	2	7	5	4	4	3	3	1	1	0	0	Heterozygote	NA	NA
15^d^	2	1	48	36	29	29	23	11	7	2	1	2	Wild type	NA	Wild type
16	1	1	19	12	9	9	7	4	3	2	1	1	Heterozygote	Heterozygote	NA
17	1	0	6	4	3	3	3	2	0	0	0	0	NA	NA	NA
18	1	1	19	10	4	4	4	3	2	2	1	1	Heterozygote	Heterozygote	NA
19	1	3	40	33	27	27	24	15	11	11	1	1	Heterozygote	NA	Heterozygote
20	1	2	25	21	13	12	10	4	4	3	1	1	Heterozygote	NA	Heterozygote
21	1	1	19	14	13	13	9	8	8	4	1	1	Wild type	Wild type	NA
22	1	1	19	13	10	10	8	5	5	3	1	1	Heterozygote	Heterozygote	NA
23	1	1	29	27	25	25	19	6	4	4	1	0	Heterozygote	NA	NA
24	1	1	16	15	15	14	10	7	4	4	1	1	Heterozygote	Heterozygote	NA
25	1	1	19	19	19	18	8	5	3	2	1	0	Wild type	Miscarriage	NA
26	1	2	8	7	4	4	4	4	4	4	1	0	Wild type	Miscarriage	NA
27	1	2	11	9	8	8	5	6	5	3	1	0	Wild type	Miscarriage	NA
28	1	1	11	11	7	7	3	3	2	1	0	0	Wild type	NA	NA
29	1	2	17	13	8	8	8	5	3	3	0	0	Wild type	NA	NA
30	2	4	47	40	33	33	16	10	7	6	2	0	Wild type	Miscarriage	NA
31^c^	2	3	27	26	14	14	8	7	6	5	1	1	Heterozygote	Heterozygote	Heterozygote
32	1	2	21	15	13	13	11	6	5	5	1	1	Heterozygote	Heterozygote	Heterozygote
33	1	0	15	14	12	12	3	3	1	0	0	0	NA	NA	NA
34	1	1	12	11	11	11	11	9	7	7	1	1	Wild type	NA	Wild type
35^c^	2	1	28	28	20	20	9	4	4	1	1	0	Heterozygote	Heterozygote	NA
36	1	1	22	17	16	15	8	6	5	2	1	0	Heterozygote	Heterozygote	NA

*PGT* preimplantation genetic testing, *FET* frozen embryo transfer, *CP* clinical pregnancy, *LB* live birth, *NA* not applicable

^a^Unaffected embryos, including non-carrier and carrier embryos

^b^Transferable embryos diagnosed as unaffected and euploid

^c^Only two biopsied blastocysts in the first cycle, and a second oocyte pick-up was performed

^d^Single blastocyst transfer which developed to monochorionic diamniotic

### NGS-based SNP haplotyping and mutation detection

Due to these families lacking relatives and probands, all analyses were based on the blastocysts. Three couples (families 8, 31, and 35) had only two biopsied blastocysts in the first cycle, and we did not perform NGS in case of impossible phasing; therefore, a second PGT cycle was performed and produced conclusive results. All of the biopsied blastocysts received conclusive PGT results (100%, 217/217) (Table 1).

**Fig. 2:**
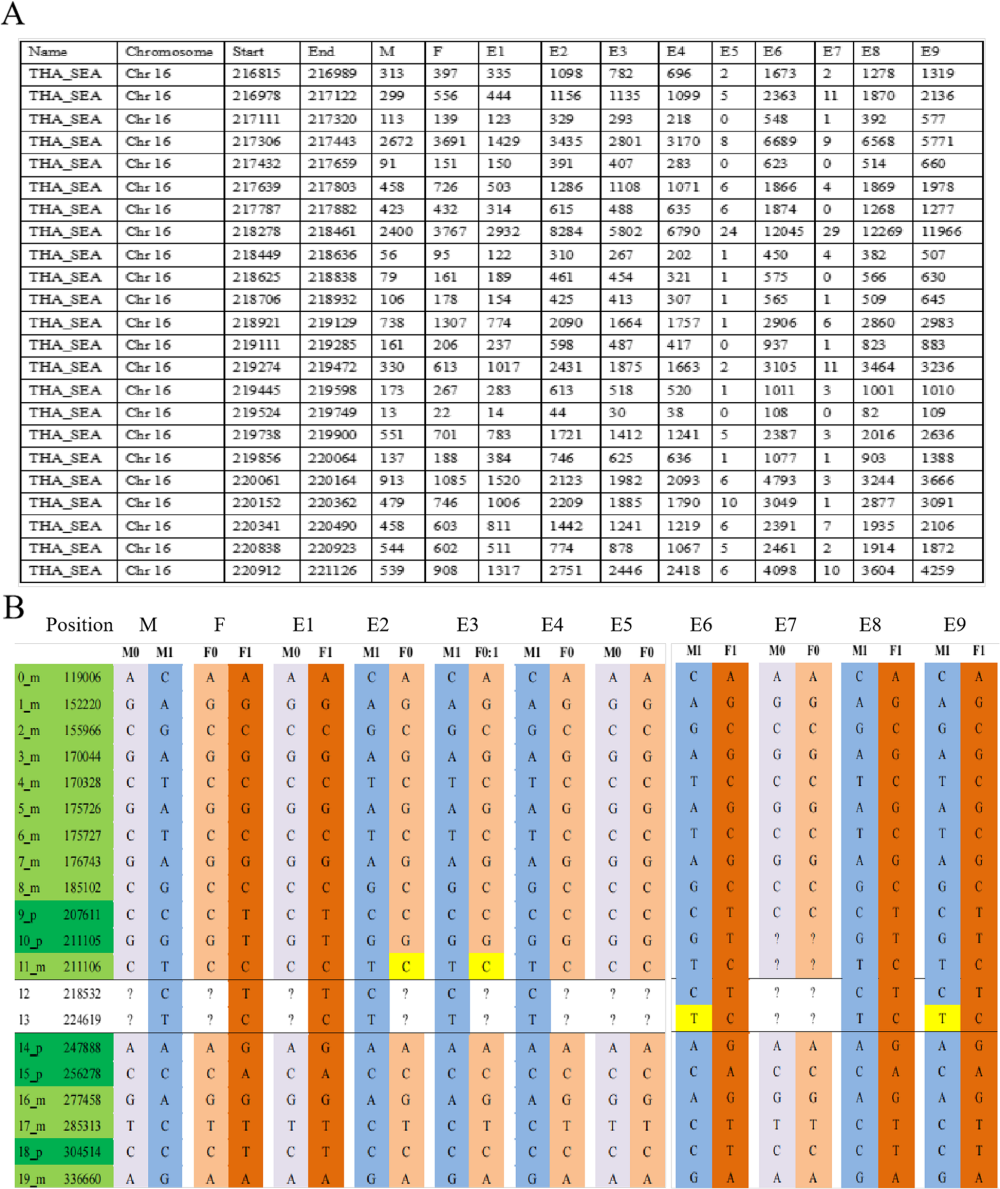
Case 13 presented as an example analysis of α- thalassemia (part of the single nucleotide polymorphism [SNP] results). **A** is a table of non-polymorphic loci that lie within the SEA deletion. Failure to amplify indicates homozygosity for the deletion. Disease bearing haplotypes were deduced from the two affected embryos (E5 and E7). The rows represent the position of the SEA deletion. Columns represent allele-specific read counts. **B** Single nucleotide polymorphisms in the SEA area were used to construct the haplotype. The positions marked in dark green are paternal informative SNPs, while those in light green are maternal informative SNPs. The yellow squares represent allele dropout sites. M0 and F0 represent an affected haplotype from the mother and father, respectively. M1 and F1 indicate the normal inherited allele. A question mark represents the undetected site in the SEA area. M: mother, F: father, E: embryo

Using family 13 as an example to analyze α-thalassemia (Fig. 2), 138 SNPs within 1 Mb upstream and 132 SNPs within 2 Mb downstream from the HBA1 and HBA2 gene were adopted with sequencing depth > 30X. We could deduce whether the mutation allele was present in the embryo by analyzing these SNPs. For instance, we found that embryos 5 and 7 carried the disease allele from the couple according to the sequencing depth of the SEA area with very low read counts (Fig. 2A, part of the SNP results). We also found that these two embryos inherited both maternal and paternal disease-associated haplotypes. Next, information SNPs in the SEA area were used to construct the haplotype. In brief, the mother was heterozygous A/C, and the father was A/A on the SNP in position 119,006. This SNP was considered as the maternal information SNP. Where the affected embryos (5 and 7) were homozygous A/A (Fig. 2B, part of the SNP results), we could easily deduce that alleles with the base A from the mother were pathogenic, and this was the disease-associated haplotype. At least two upstream and two downstream markers closely linked to the gene underlying the mutation were analyzed, and the disease-associated and non-disease-associated maternal haplotype was successfully distinguished. Hence, we concluded that embryos 1, 5, and 7 carried the disease-associated maternal haplotype. Similarly, in position 207,611, where the mother was C/C, and the father was C/T, this SNP was considered as the paternal information SNP (Fig. 2B). Where the affected embryos (5 and 7) were homozygous C/C, we could easily deduce that alleles with base C from the father were pathogenic, and this was another disease-associated haplotype. Hence, we concluded that embryos 2, 3, 4, 5, and 7 carried the disease-associated paternal haplotype. So, embryos 5 and 7 were homozygous, embryos 1, 2, 3, and 4 were heterozygous, and embryos 6, 8, and 9 were wildtype.

**Fig. 3:**
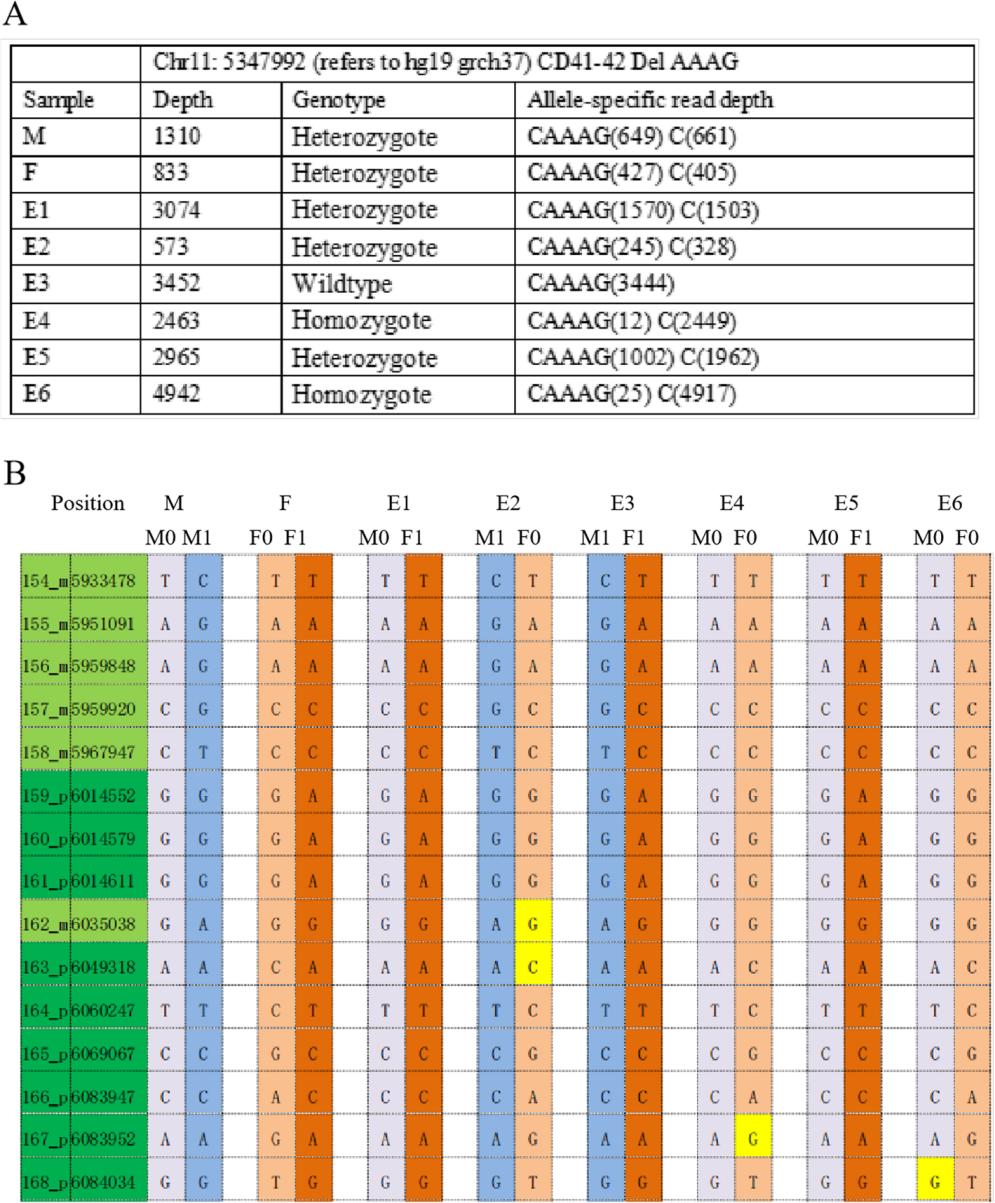
Case 30, presented as an example analysis of β- thalassemia, both of the parents carry the same mutation (del AAAG) in the CD 41–42 area. **A**. Sequencing depth of the CD 41–42 area. **B**. Single nucleotide polymorphisms (SNPs) near the CD 41–42 area were used to construct the haplotype (part of the SNP results). The positions marked in dark green are paternal informative SNPs while the positions in light green are maternal informative SNPs. The yellow squares represent allele dropout sites. M0 and F0 represent an affected haplotype from the mother and father, respectively. M1 and F1 indicate the normal inherited allele. M: mother, F: father, E: embryo. CD41–42DelAAAG: *HBB* gene condon (CD) 41–42 mutation (− 4 bp, AAAG)

The analysis method for β- thalassemia (using family 30 as an example) is shown in Fig. 3. Ninety-five SNPs within 2 Mb upstream and downstream respective from the *HBB* gene were adopted with sequencing depth > 30X. At first, we could deduce embryos 4 and 6 carried the disease allele according to the sequencing depth of the βCD41–42 (Fig. 3A). Thus, we deduced that these two embryos inherited both maternal and paternal disease-associated haplotypes (Fig. 3B). Next, information SNPs in the *HBB* were used to construct the haplotype as described above. We could also conclude that embryos 1, 4, 5, and 6 carried the disease-associated maternal haplotype and that embryos 2, 4, and 6 carried the disease-associated paternal haplotype. So, embryos 4 and 6 were homozygous, embryos 1, 2, and 5 were heterozygous, and embryo 3 was wildtype.

### PGT results

After NGS-based SNP haplotyping and mutation detection, 160 (73.7%, 160/217) blastocysts were found to be unaffected by either α- thalassemia or β- thalassemia (Table 1). All of the results were confirmed by Sanger sequencing or conventional PCR. Preimplantation genetic testing for aneuploidy results of these blastocysts showed that 112 (70.0%, 112/160) were euploid, which were defined as transferable blastocysts (Table 1). The average number of transferable blastocysts for each couple was 3.1 (112/36). The rate of blastocyst mosaicism was 10.6% (23/217). Because couples with mosaic blastocysts had normal blastocysts to transfer, none of these mosaic blastocysts were transferred in this study.

### Clinical outcomes

Two couples had no transferable blastocysts after the first PGT cycle, and did not perform another PGT cycle. In the other 34 couples, a single blastocyst was transferred to the uterus (53 FET cycles). Thirty-two cycles resulted in clinical pregnancy, and the clinical pregnancy rate was 60.1% (32/53) per FET cycle. Family 15 developed a monochorionic diamniotic twin pregnancy after a single blastocyst transfer, resulting in the birth of two healthy babies.

Twenty-two cycles (22 couples) resulted in 23 live births, and the live birth rate was 43.4% (23/53, 3 cycles were ongoing pregnancies). The prenatal diagnosis results and/or thalassemia gene analyses after the delivery were concordant with the NGS-PGT results for all 25 cycles. Seven cycles resulted in miscarriage before 12 weeks’ gestation (7/32, 21.9%), and the abortion villus from four of the cycles showed normal karyotype and thalassemia results consistent with the NGS-PGT results. However, samples from the aborted fetuses in 3 cycles were not available because the pregnancy lasted less than 5 weeks.

## Discussion

The small amount of DNA taken from biopsied trophectoderm cells and amplification bias based on WGA can lead to ADO [16]. Polymerase chain reaction-based methods for PGT are inevitably affected by ADOs. Although haplotype analysis with short tandem repeat (STR) may reduce the effects of ADO, the number of STR loci is limited. Further, recombination between STR loci and target genes may affect the diagnostic accuracy [17, 18]. Hence, single-nucleotide polymorphisms (SNPs) linked to the mutated genes are increasingly being used to establish haplotype linkages in clinical practice [19]. However, this technique requires probands or the pedigrees of the parents to construct the haplotype for linkage analysis. This makes performing PGT difficult for detecting some inherited genetic diseases like α-thalassemia.

At present, the most widely used method for PGT-M without pedigrees is single-sperm-based SNP haplotyping, namely, isolating a single sperm cell and analyzing the genotypes of SNP alleles using NGS [10, 20]. However, polymorphic markers need to be identified before linkage analysis, and this requires multiple steps and extra laboratory work. This method is usually suitable for paternally-inherited dominant diseases, such as Osteogenesis imperfecta [10]. However, both α-thalassemia and β-thalassemia are recessive inherited diseases. Thus, using single-sperm-based SNP haplotyping would be tedious and could not identify the maternal haplotype unless polar bodies are also biopsied.

Next-generation sequencing-based PGT can simultaneously detect target mutation sites and linked SNPs, making it possible to provide multiple diagnostic results with the advantages of high accuracy and throughput [21]. Therefore, we chose to use affected embryos as a reference to perform haplotype construction in order to avoid the multiple steps. All biopsied blastocysts yielded conclusive PGT results. This method is very useful in a clinic setting as many inherited monogenetic diseases lack intact pedigrees, such as in the case of our previous case report PGT-M for Marfan syndrome [12]. A previous study by Ren et al. [11] successfully carried out PGT-M based on a mutated allele by sequencing with aneuploidy and linkage analyses for two carrier families with children affected with spinal muscular atrophy. They found that this method could correctly diagnose embryos by using affected embryos as the probands. Another study by Chen et al. [4] also found that NGS-based haplotyping could be performed by directly detecting mutation sites and using affected embryos as probands for PGT-M. Li et al. [22] successfully applied the linked-read sequencing method to construct parental haplotypes without recruiting additional family members in two families with alpha thalassemia and in one with NDP gene disorder. However, these studies only included one or two couples. Therefore, it is necessary to conduct studies with more samples to validate the use of affected embryos as probands in a clinic setting.

In this study, 29 couples with α-thalassemia and seven couples with β-thalassemia successfully underwent PGT using affected embryos as a reference. All 217 biopsied blastocysts yielded conclusive PGT results. After a single blastocyst was transferred in 53 cycles, 32 cycles resulted in clinical pregnancy, and 22 cycles (23 babies) resulted in a live birth. Unfortunately, the miscarriage rate for euploid embryos was high in our study. Since thalassemia itself does not cause miscarriage, the suspected reasons for this high miscarriage rate were the biopsy procedure or blastocyst quality. All prenatal diagnosis results and/or thalassemia gene analyses after delivery were concordant with the NGS-PGT results. Therefore, we successfully conducted PGT for thalassemia using SNP haplotyping with affected embryos as a reference. This technique is very useful for some couples with other monogenetic diseases who need to perform PGT without probands and parental pedigrees.

Although there are challenges associated with ADO, haplotyping linkage analysis with more informative SNPs could help to avoid this. Next-generation sequencing-based PGT can detect the mutated gene directly and construct haplotypes with SNPs close to the mutated gene using PGT to determine ADO and prevent misdiagnosis. Moreover, haplotyping can also be used to distinguish the chromosome of the pathogenic gene from normal chromosomes and find monosomies of chromosomes to avoid misdiagnoses. In our study, more than 100 SNP markers within 1 Mb upstream and downstream of the pathogenic mutation site were used to establish the haplotype. By analyzing these SNPs, we could determine the disease-carrying allele state of each embryo. However, having an affected embryo that allows haplotype phasing was a matter of probability. Even with 8 embryos, there was still a 10% chance that none were affected. And patients must start the PGT process with no guarantee that it can be completed and they need adequate explanation and counseling about this possibility.

The main limitation of this method is that patients may not obtain sufficient embryos as a reference, requiring another oocyte pick-up cycle. Additionally, marriages in proximity may not lead to enough informative SNPs to establish haplotyping.

## Conclusion

Next-generation sequencing can be used to conduct PGT for thalassemia using affected embryos as a reference. Additionally, this method could also be used to perform PGT for other monogenic diseases in the absence of probands and parental pedigrees.

## Supplementary Information

**Additional file 1: Supplemental Table 1.** The demographic information of the 36 families. Reproductive history: G: gravidity, P: parity, A: abortion, EP: ectopic pregnancy. Induced labor for severe thalassemia: numbers indicate how many times labor was induced for severe thalassemia before PGT. Probands: NO: never pregnant; NA: not applicable, meaning the blood for haplotyping could not obtained.

## Data Availability

Data were obtained from the referenced publications. For further information, contact Dr. Ou at zhanhui-ou@hotmail.com.
