# Supplementary material for: Using affected embryos to establish linkage phase in preimplantation genetic testing for thalassemia
Source: Reprod Biol Endocrinol. 2022 Apr 30;20:75. doi: 10.1186/s12958-022-00948-9 (PMC9055750; doi:10.1186/s12958-022-00948-9)
Supplement: Supplementary file 1 — Additional file 1: Supplemental Table 1. The demographic information of the 36 families. Reproductive history: G: gravidity, P: parity, A: abortion, EP: ectopic pregnancy. Induced labor for severe thalassemia: numbers indicate how many times labor was induced for severe thalassemia before PGT. Probands: NO: never pregnant; NA: not applicable, meaning the blood for haplotyping could not obtained. [file 12958_2022_948_MOESM1_ESM.doc]

Supplemental table 1 The demographic information of the 36 families.

| Family | Age | | Reproductive history | Induced labors for severe thalassemia | Proband | Thalassemia mutations | |
| --- | --- | --- | --- | --- | --- | --- | --- |
|  | Maternal | Paternal |  |  |  | Maternal | Paternal |
| 1 | 35 | 34 | G0P0A0EP0 | 0 | No | --SEA/αα | --SEA/αα |
| 2 | 29 | 33 | G0P0A0EP0 | 0 | No | --SEA/αα | --SEA/αα |
| 3 | 37 | 37 | G1P0A1EP0 | 0 | No | --SEA/αα | --SEA/αα |
| 4 | 32 | 32 | G1P0A1EP0 | 0 | No | --SEA/αα | --SEA/αα |
| 5^1^ | 27 | 32 | G2P0A2EP0 | 1 | NA | --SEA/αα | --SEA/αα |
| 6 | 31 | 31 | G1P0A1EP0 | 1 | NA | --SEA/αα | --SEA/αα |
| 7 | 26 | 29 | G1P0A0EP1 | 0 | No | --SEA/αα | -α4.2/αα |
| 8 | 30 | 36 | G1P0A1EP0 | 0 | No | --SEA/αα | --SEA/αα |
| 9 | 24 | 26 | G1P0A1EP0 | 1 | NA | --SEA/αα | --SEA/αα |
| 10 | 29 | 37 | G0P0A0EP0 | 0 | No | --SEA/αα | --SEA/αα |
| 11 | 35 | 36 | G2P0A3EP0 | 1 | NA | --SEA/αα | --SEA/αα |
| 12 | 27 | 27 | G0P0A0EP0 | 0 | No | --SEA/αα | --SEA/αα |
| 13 | 26 | 27 | G0P0A0EP0 | 0 | No | --SEA/αα | --SEA/αα |
| 14 | 24 | 28 | G0P0A0EP0 | 0 | No | --SEA/αα | --SEA/αα |
| 15 | 30 | 31 | G0P0A0EP0 | 0 | No | --SEA/αα | --SEA/αα |
| 16 | 30 | 32 | G0P0A0EP0 | 0 | No | --SEA/αα | -a3.7/--SEA |
| 17 | 31 | 32 | G1P0A1EP0 | 0 | No | --SEA/αα | -a3.7/--SEA |
| 18 | 31 | 35 | G1P0A1EP0 | 1 | NA | -a3.7/--SEA | --SEA/αα |
| 19 | 25 | 25 | G0P0A0EP0 | 0 | No | αWS/αα | --SEA/αα |
| 20 | 32 | 35 | G3P0A3EP0 | 0 | No | -a3.7/αα | --SEA/αα |
| 21 | 34 | 33 | G1P0A1EP0 | 0 | NA | --SEA/αα | -a3.7/αα |
| 22 | 31 | 35 | G0P0A0EP0 | 0 | No | --SEA/αα | -a3.7/αα |
| 23 | 28 | 23 | G2P0A2EP0 | 1 | NA | --SEA/aa^WS^ | --SEA/αα |
| 24 | 27 | 33 | G3P0A3EP0 | 2 | NA | -a3.7/--SEA | --SEA/αα |
| 25 | 33 | 35 | G0P0A0EP0 | 0 | No | --SEA/αα | --SEA/αα |
| 26 | 30 | 31 | G0P0A0EP0 | 0 | No | --SEA/αα | --SEA/αα |
| 27 | 34 | 33 | G1P0A1EP0 | 0 | No | -a3.7/αα | --SEA/αα |
| 28 | 37 | 43 | G2P0A2EP0 | 1 | NA | --SEA/αα | --SEA/αα |
| 29 | 24 | 28 | G0P0A0EP0 | 0 | No | --SEA/αα | --SEA/αα |
| 30 | 27 | 25 | G0P0A0EP0 | 0 | No | βCD41-42/βN | βCD41-42/βN |
| 31 | 27 | 35 | G0P0A0EP0 | 0 | No | βCD41-42/βN | βCD41-42/βN |
| 32 | 28 | 31 | G2P0A2EP0 | 2 | NA | βCD41-42/βN | βCD41-42/βN |
| 33 | 32 | 32 | G1P0A1EP0 | 1 | NA | β-28/β-N | βCD41-42/βN |
| 34 | 30 | 32 | G0P0A0EP0 | 0 | No | β-654/βN | β-654/βN |
| 35 | 39 | 43 | G1P0A0EP1 | 0 | No | βCD41-42/βN | β-28/β-N |
| 36 | 26 | 28 | G1P0A1EP0 | 1 | NA | βCD17/βN | βCD41-42/βN |

G: gravidity, P: parity, A: abortion, EP: ectopic pregnancy

1 Maternal both with a-thalassemia and chromosomal balanced translocations.

No: never pregnancy; NA: not applicable, mean the blood for haplotyping could not obtained.
